# Supplementary material for: Quantitative evaluation of Mycobacterium abscessus clinical isolate virulence using a silkworm infection model
Source: PLoS One. 2022 Dec 20;17(12):e0278773. doi: 10.1371/journal.pone.0278773 (PMC9767372; doi:10.1371/journal.pone.0278773)
Supplement: Attachment — Submitted filename: Response to reviewers comment.doc [file pone.0278773.s002.doc]

Editor’s comment

1. Please ensure that your manuscript meets PLOS ONE's style requirements, including those for file naming. The PLOS ONE style templates can be found at

https://journals.plos.org/plosone/s/file?id=wjVg/PLOSOne_formatting_sample_main_body.pdf and https://journals.plos.org/plosone/s/file?id=ba62/PLOSOne_formatting_sample_title_authors_affiliations.pdf

According to the editor’s comment, we revised the manuscript based on the PLOS ONE style templates.

2. Please provide additional details regarding participant consent. In the ethics statement in the Methods and online submission information, please ensure that you have specified (1) whether consent was informed and (2) what type you obtained (for instance, written or verbal, and if verbal, how it was documented and witnessed). If your study included minors, state whether you obtained consent from parents or guardians. If the need for consent was waived by the ethics committee, please include this information.

If you are reporting a retrospective study of medical records or archived samples, please ensure that you have discussed whether all data were fully anonymized before you accessed them and/or whether the IRB or ethics committee waived the requirement for informed consent. If patients provided informed written consent to have data from their medical records used in research, please include this information

Once you have amended this/these statement(s) in the Methods section of the manuscript, please add the same text to the “Ethics Statement” field of the submission form (via “Edit Submission”).

For additional information about PLOS ONE ethical requirements for human subjects research, please refer to http://journals.plos.org/plosone/s/submission-guidelines#loc-human-subjects-research.

Thank you very much for the suggestion. Because we did not use the patient information in this study, informed consent was not needed.

3. Thank you for stating the following in the Acknowledgments Section of your manuscript:

“This study was 328 supported in part by grants from the Japan Agency for Medical Research and Development/Japan International Cooperation Agency (AMED) to YH (JP20fk0108064, JP20fk0108075, JP21fk0108093, JP21fk0108129, JP21fk0108608, JP21jm0510004, JP21wm0125007, JP21wm0225004, JP21wm0325003, JP22gm1610003, JP22wm0225022, JP22wm0325054), to HF (JP22wm0325054), and Y.M. (JP22wm0325054); and for Scientific Research (C) to YM (JP20K07022) from the Japan Society for the Promotion of Science (JSPS).The funders had no role in the study design, data collection, data analysis, decision to publish, or preparation of the manuscript.”

We note that you have provided additional information within the Acknowledgements Section that is not currently declared in your Funding Statement. Please note that funding information should not appear in the Acknowledgments section or other areas of your manuscript. We will only publish funding information present in the Funding Statement section of the online submission form.

Please remove any funding-related text from the manuscript and let us know how you would like to update your Funding Statement. Currently, your Funding Statement reads as follows:

“This study was supported in part by grants from the Japan Agency for Medical Research and Development/Japan International Cooperation Agency (AMED) to YH (JP20fk0108064, JP20fk0108075, JP21fk0108093, JP21fk0108129, JP21fk0108608, JP21jm0510004, JP21wm0125007, JP21wm0225004, JP21wm0325003, JP22gm1610003, JP22wm0225022, JP22wm0325054), to HF (JP22wm0325054), and Y.M. (JP22wm0325054); and for Scientific Research (C) to YM (JP20K07022) from the Japan Society for the Promotion of Science (JSPS). The funders had no role in the study design, data collection, data analysis, decision to publish, or preparation of the manuscript.”

Please include your amended statements within your cover letter; we will change the online submission form on your behalf.

According to the editor’s comment, we removed the funding-related text in the revised manuscript.

Moreover, the Funding Statement was described below.

This study was supported in part by grants from the Japan Agency for Medical Research and Development/Japan International Cooperation Agency (AMED) to YH (JP20fk0108064, JP20fk0108075, JP21fk0108093, JP21fk0108129, JP21fk0108608, JP21jm0510004, JP21wm0125007, JP21wm0225004, JP21wm0325003, JP22gm1610003, JP22wm0225022, JP22wm0325054), to HF (JP22wm0325054), and Y.M. (JP22wm0325054); and for Scientific Research (C) to YM (JP20K07022) from the Japan Society for the Promotion of Science (JSPS). The funders had no role in the study design, data collection, data analysis, decision to publish, or preparation of the manuscript.

4. Please review your reference list to ensure that it is complete and correct. If you have cited papers that have been retracted, please include the rationale for doing so in the manuscript text, or remove these references and replace them with relevant current references. Any changes to the reference list should be mentioned in the rebuttal letter that accompanies your revised manuscript. If you need to cite a retracted article, indicate the article’s retracted status in the References list and also include a citation and full reference for the retraction notice.

We have checked.

Reviewer #1: A nice study, easy to follow. I have only two text addition requests for the discussion and one statistical recalculation:

How accurate is hemolymph for assessing MABC burden? Presumably these bacteria can invade the tissue like they do in other model systems so there might be unaccounted burden missed by a more thorough method such as homogenisation. Please elaborate discussion to speculate if tissue invasion is expected and if the silk worm in amenable to histological study.
According to the reviewer’s comment, we added the sentences in the Discussion section of the revised manuscript (Page 15, lines 324-329).

[Page 15, lines 324-329]

Our study has some limitations. First, the silkworm infection model established in this study is not a respiratory infection model because the bacterial cells were injected into silkworm hemolymph. Therefore, the silkworm infection model deviates significantly from *M. abscessus* infection in humans. Second, it is unknown the reason to cause silkworm death by growing the bacteria in which tissues of the silkworm. Therefore, histological studies need to determine silkworm tissue which is more abundant bacterial cells.

Line 308: zebrafish models can evaluate virulence < 7 days (Kremer lab) in embryos, or the 14-15 day time period is from an Oehlers lab paper using adult zebrafish. It is worth commenting around this paragraph on the temperature differences, the silk worm allows the study of virulence at 37 while zebrafish studies are in the 28-32 degree range.
According to the reviewer’s comment, we added the sentence that the rearing temperature of the animals affects the experimental period.

[Page 15, lines 313-316]

The rearing temperature may be related to the experimental period. For example, infected zebrafishes were reared at 28-32˚C. In this study, infected silkworms died under a rearing condition at 37˚C within 2 days but not at 27˚C. Therefore, the silkworm has the benefit of being able to examine the infection experiments at 37˚C.

Figure 7: please replace T test with ANOVA

Following the reviewer’s comment, we performed the Turkey test with one-way ANOVA in Figure 7 (Page 13, line 273).

[Page 13, line 273]

using the Turkey test with one-way ANOVA

Reviewer #2: Virulence of seven clinical isolates of Mycobacterium abscessus subspecies abscessus was investigated using silkworm infection model. Additional, using a standard/validated protocol, virulence of these isolates was also studied in human macrophage cell line THP-1. Overall, the studies were straightforward and systematically performed. Data were interpreted in a rigorous manner and conclusions are justified by the results. Below I have included recommendations for revisions to improve on clarity and accuracy. Also, I have recommended to include additional discussion on the limitations of these M. abscessus infection model.
According to the reviewer’s comment, we added the limitations in the new paragraph of the Discussion section (Page 15, lines 324-329).

[Page 15, lines 324-329]

Our study has some limitations. First, the silkworm infection model established in this study is not a respiratory infection model because the bacterial cells were injected into silkworm hemolymph. Therefore, the silkworm infection model deviates significantly from *M. abscessus* infection in humans. Second, it is unknown the reason to cause silkworm death by growing the bacteria in which tissues of the silkworm. Therefore, histological studies need to determine silkworm tissue which is more abundant bacterial cells.

MINOR COMMENTS:
1. Abstract: It is redundant to use M. abscessus subspecies M. abscessus throughout the abstract as only supspecies abscessus is used in the study. So, please delete ‘subspecies abscessus’ from the following lines (Line 25, another one in line 25, line 26, 27, 29).

Following the reviewer’s comment, we delete the description “subspecies abscessus” in the Abstract section of the revised manuscript.

2. Line 28: add ‘in humans’ after ‘infection’.

Following the reviewer’s comment, we added the description in the revised manuscript (Page 2, line 26).

3. Line 30: replace ‘were determined using’ with ‘in’.

Following the reviewer’s comment, we replaced the description in the revised manuscript (Page 2, line 28).

4. Line 33: delete ‘with’.

Following the reviewer’s comment, we delete the description in the revised manuscript.

5. Line 46: replace ‘effects’ with ‘efficacies’.

Following the reviewer’s comment, we replaced the description in the revised manuscript (Page 3, line 44).

6. Line 46-48: It is stated that ‘mice infected with MABC dies over several months’. This is completely untrue. Even the cited references show that it requires immunesuppression or immunecompromised mice to maintain MABC infection. So this sentence is factually incorrect and should be deleted. Replace it with the following sentence: ‘Existing mouse models of MABC infection require several weeks to complete a single study, which is not convenient especially for MABC virulence screening purposes, and thus the development of a model that permits a more rapid evaluation of MABC virulence is highly desirable.’

Thank you for pointing this out and kind suggestion. According to the reviewer’s suggestion, we deleted the sentence and added the suggested sentence in the revised manuscript (Page 3, lines 44-47).

[Page 3, lines 44-47]

Existing mouse models of MABC infection require several weeks to complete a single study, which is not convenient, especially for MABC virulence screening purposes. Thus, developing a model that permits a more rapid evaluation of MABC virulence is highly desirable.

7. Animal models of a human disease have their strengths and limitations. A good and objective manuscript should include limitations of the model. The authors should include the fact that most of the Mycobacterium abscessus infections are acquired via aerosol and this model does not take that into account. Most of the Mycobacterium abscessus disease is pulmonary and this model cannot recapitulate that aspect. The formation of lesion in the lungs and immune response are distinct from that in the silkworm. Therefore, it is important to include limitations of the silkworm model. Then describe the unique strengths of this model, which is the rapid time in which virulence of M. abscessus can be studied. Also, a large number of silkworms can be used, which is important for screening studies.

Thank you for your kind suggestion. According to the reviewer’s suggestion. We added the sentence of the limitations in this study of the Discussion section in the revised manuscript (Page 15, lines 324-329).

[Page 15, lines 324-329]

Our study has some limitations. First, the silkworm infection model established in this study is not a respiratory infection model because the bacterial cells were injected into silkworm hemolymph. Therefore, the silkworm infection model deviates significantly from *M. abscessus* infection in humans. Second, it is unknown the reason to cause silkworm death by growing the bacteria in which tissues of the silkworm. Therefore, histological studies need to determine silkworm tissue which is more abundant bacterial cells.

8. NOTE that the study assesses ‘virulence’ and not ‘pathogenecity’ of M. abscessus isolates.

Thank you very much for your suggestion. We revised the manuscript according to the reviewer’s comment (Page 11, line 227, 236).

9. Figure 1: what is n? n should be described as the number of independent M. abscessus isolates so that it is not confused with the number of silkworms used. Also, the number of silkworms used should be included in the figure legend. Pathogenecity study should include progression of disease in terms of tissue pathology and disease phenotypes in the host as the disease evolves. Therefore, ‘virulence’ is the correct terms and ‘pathogenecity’ should not be used. Line 31: replace ‘pathogenic’ with ‘virulent’. Line 224: replace ‘pathogenecity’ with ‘virulence’. Line 233: replace ‘pathogenecity’ with ‘virulence’. Line 295: replace ‘pathogenic’ with ‘virulent’.

According to the reviewer’s comment, we replaced the words in the revised manuscript. Moreover, we changed the Figure legends of the revised manuscript (Page 9, lines 180, 183-184, Page 11, line 227, 236).

10. Figure 2. In the figure legend ‘n’ should be defined as the number of silkworms used.

Following the reviewer’s comment, we described the number of silkworms used (Page 9, lines 190-191).

11. Figure 3. What is ‘n’? Is it the number of silkworms used or M. abscessus isolates?

Following the reviewer’s comment, we described the number of silkworms used (Page 10, lines 212-213).

12. Figure 4: Please define ‘n’. It represents the number of what?

Following the reviewer’s comment, we described the number of silkworms used (Page 11, lines 219-220, 225).

13. Figure 5: Please define ‘n’.

Following the reviewer’s comment, we described the number of silkworms used (Page 12, line 244).

14. Figure 6: Please define ‘n’.

Following the reviewer’s comment, we described the number of silkworms used (Page 12, lines 251-252).

15. Line 97: insert ‘were’ after ‘silkworms’.

Following the reviewer’s comment, we added the word in the revised manuscript (Page 5, line 98).

16. Line 98: insert ‘ , and’ after ‘larva)’.

Following the reviewer’s comment, we added the word in the revised manuscript (Page 5, line 99).

17. Line 99: Briefly describe how silkworms were injected with M. abscessus. What type of instrument/device was used to deliver M. abscessus?

According to the reviewer’s comment, we added the sentence in the revised manuscript (Page 5, lines 96-98).

[Page 5, lines 96-98]

A 50-μl of sample solutions was administered to the silkworm hemolymph by injecting the silkworm dorsally using a 1-ml tuberculin syringe (Terumo Medical Corporation, Tokyo, Japan).

18. Line 200: Insert ‘in humans’ after ‘infections’.

Following the reviewer’s comment, we added the word in the revised manuscript (Page 10, line 202).

19. Line 288: replace ‘severe’ with ‘severity’.

Following the reviewer’s comment, we replaced the word in the revised manuscript (Page 14, line 292).

20. Line 299: replace ‘was identified as a highly pathogenic strain using the’ with ‘is highly virulent in’

Following the reviewer’s comment, we replaced the sentence in the revised manuscript (Page 14, line 303).

21. Line 303: delete ‘avirulent’. This term is unnecessary here.

Following the reviewer’s comment, we deleted the word in the revised manuscript.

22. Line 305: The cited reference #33 by Foreman et al is not appropriate here as random transposon mutagenesis is described in the publication rather than targeted gene deletion in MABC. This reference should be removed. More relevant references are [Nessar R, Reyrat JM, Davidson LB, Byrd TF. Deletion of the mmpL4b gene in the Mycobacterium abscessus glycopeptidolipid biosynthetic pathway results in loss of surface colonization capability, but enhanced ability to replicate in human macrophages and stimulate their innate immune response. Microbiology (Reading). 2011 Apr;157(Pt 4):1187-1195. doi: 10.1099/mic.0.046557-0. Epub 2011 Feb 3. PMID: 21292749.] and [Galanis C, Maggioncalda EC, Kumar P, Lamichhane G. Glby, Encoded by MAB_3167c, Is Required for In Vivo Growth of Mycobacteroides abscessus and Exhibits Mild β-Lactamase Activity. J Bacteriol. 2022 May 17;204(5):e0004622. doi: 10.1128/jb.00046-22. Epub 2022 Apr 5. PMID: 35380462; PMCID: PMC9112878.].

Thank you very much for your suggestion. According to the reviewer’s comment, we deleted the reference 33 in the previous manuscript and added the new references in the revised manuscript (Page 15, line 308, pages 21-22, lines 442-446, page 22, lines 451-454).

23. Line 308: insert ‘mice’ after ‘Danio rerio’. Mice should be included in this list, otherwise this sentence will be factually incorrect.

Following the reviewer’s comment, we added the word in the revised manuscript (Page 15, line 331).
